# Thiophene-Based Compounds with Potential Anti-Inflammatory Activity

**DOI:** 10.3390/ph14070692

**Published:** 2021-07-19

**Authors:** Ryldene Marques Duarte da Cruz, Francisco Jaime Bezerra Mendonça-Junior, Natália Barbosa de Mélo, Luciana Scotti, Rodrigo Santos Aquino de Araújo, Reinaldo Nóbrega de Almeida, Ricardo Olímpio de Moura

**Affiliations:** 1 Post-Graduation Program in Natural and Synthetic Bioactive Products, Federal University of Paraíba, João Pessoa 58051-900, PB, Brazil; ryldene@hotmail.com (R.M.D.d.C.); luciana.scotti@gmail.com (L.S.); reinaldoan@uol.com.br (R.N.d.A.); 2 Laboratory of Synthesis and Drug Delivery, State University of Paraíba, João Pessoa 58071-160, PB, Brazil; nataliamelo926@hotmail.com (N.B.d.M.); rodrigobiologojp@gmail.com (R.S.A.d.A.); ricardo.olimpiodemoura@gmail.com (R.O.d.M.)

**Keywords:** inflammation, thiophene, molecular docking, cyclooxygenase, lipoxygenase, anti-inflammatory drugs

## Abstract

Rheumatoid arthritis, arthrosis and gout, among other chronic inflammatory diseases are public health problems and represent major therapeutic challenges. Non-steroidal anti-inflammatory drugs (NSAIDs) are the most prescribed clinical treatments, despite their severe side effects and their exclusive action in improving symptoms, without effectively promoting the cure. However, recent advances in the fields of pharmacology, medicinal chemistry, and chemoinformatics have provided valuable information and opportunities for development of new anti-inflammatory drug candidates. For drug design and discovery, thiophene derivatives are privileged structures. Thiophene-based compounds, like the commercial drugs Tinoridine and Tiaprofenic acid, are known for their anti-inflammatory properties. The present review provides an update on the role of thiophene-based derivatives in inflammation. Studies on mechanisms of action, interactions with receptors (especially against cyclooxygenase (COX) and lipoxygenase (LOX)), and structure-activity relationships are also presented and discussed. The results demonstrate the importance of thiophene-based compounds as privileged structures for the design and discovery of novel anti-inflammatory agents. The studies reveal important structural characteristics. The presence of carboxylic acids, esters, amines, and amides, as well as methyl and methoxy groups, has been frequently described, and highlights the importance of these groups for anti-inflammatory activity and biological target recognition, especially for inhibition of COX and LOX enzymes.

## 1. Introduction

Inflammation is a tightly and carefully regulated, protective process, both complex and multifactorial, and mounted by the innate immune system in response to harmful stimuli such as ischemia, tissue damage, autoimmune injuries, dead cells, pathogens, toxins, and chemicals. The mechanisms involved in this process are characterized by a complex series of events that involve changes in vascular permeability, exudation of fluids containing plasma proteins, and migration cells within the immune system, such as leukocytes, lymphocytes, and macrophages into the inflammatory area [1,2,3,4,5].

These mechanisms are mediated through a great variety of soluble micromolecules, which include several secreted polypeptides known as cytokines. Inflammatory cytokines can be classified and are involved in both acute and chronic inflammation. In accordance with the cellular microenvironment, they may present either pro- (Th1) or anti- (Th2) inflammatory activities. The most common anti-inflammatory cytokines are interleukins IL-4, IL-10, IL-13, and TGFβ (transforming growth factor). The most common proinflammatory cytokines are tumor necrosis factor (TNF), and interleukins IL-1, IL-2, IL-6 and IL-7 [5,6,7,8].

To maintain or to re-establish homeostasis, proinflammatory cytokine control, production, and regulation are essential. Controlled proinflammatory cytokine production helps contain the inflammatory process and reduce tissue damage. However, excess production exacerbates the inflammatory process, in many cases generating the onset of chronic inflammatory disease [7,9,10].

The current clinical treatment options for inflammatory diseases are based on the use of non-steroidal anti-inflammatory drugs (NSAIDs) and steroids. Despite causing several known severe side effects, including cardiovascular, renal, and hepatic disorders, and above all gastrointestinal toxicity, NSAIDs remain the most prescribed drugs in medicine [11,12,13,14,15]. Many of their side effects are due to their mechanisms of action, being associated with inhibition of the enzymes cyclooxygenase (COX) and lipoxygenase (LOX), which directly mediate the production of prostaglandins and leukotrienes, many of which provide protective functions in the body [16,17,18].

In this context, about one in four people in the world is affected by some chronic pain arising from diseases of inflammatory origin, and these patients seek health services more frequently than the rest of the population. Inflammatory diseases are important factor involved in mortality and morbidity in humans; they are also a public health problem [19,20,21]. One of the great current challenges is to develop more effective and safe NSAIDs, as well as other therapeutic alternatives to treat inflammatory conditions, especially chronic inflammatory diseases [4,22].

Heterocyclic compounds have historically played an important role in the search for bioactive products. It is observed that more than 75% of drugs in clinical use have at least one heterocyclic ring in their chemical structure [23]. Thiophene and its substituted derivatives, all heterocyclic compounds, have been our focus of interest for almost two decades.

Thiophene derivatives provide useful intermediaries in various areas of science and industry, with a wide range of applications, and therapeutic properties. Thiophene derivatives attract both great academic interest, and interest from the agrochemical, pharmaceutical, and dye industries, as well [5,24,25]. As to their biological and pharmacological applications, thiophene derivatives possess remarkable properties as antipsychotic, antianxiety, antifungal, antimicrobial, antioxidant, anticancer, and anti-inflammatory agents [5,24,26,27,28,29]. Many marketed drugs, such as Olanzapine, Benzocyclidine, Sertaconazole, Tioconazol, Dorzolamide, Tipepidine, Ticlopidine, Clopidogrel, Pasugrel, Citizolam, Timepidium and Tiquizium Bromide contain a thiophene moiety.

**Figure 1:**
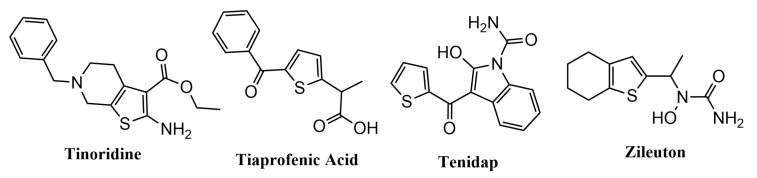
Chemical structures of market anti-inflammatory drugs containing a thiophene moiety (Tinoridine, Tiaprofenic acid, Tenidap and Zileuton.).

Tinoridine, Tiaprofenic acid, Tenidap, and Zileuton (Figure 1) are the best-known examples of commercially available drugs with anti-inflammatory properties that contain a thiophene ring as pharmacophoric group. The first three are NSAIDs used in the treatment of pain and inflammation. Tinoridine and Tiaprofenic acid act by inhibiting COX enzymes [26,30], and Tinoridine presents potent antiperoxidative and radical scavenger activity [30], while Zileuton is a LOX inhibitor [31].

Based on these initial considerations, the aim of this review is to present an update over the last 10 years of the role of thiophene derivatives in inflammation, identify the most promising compounds and anti-inflammatory substitution patterns, and to help direct the synthesis of potentially more active new derivatives. To compose the database, the authors performed a systematic search in PubMed, Capes Journal Portal, ScieELO and Medline databases. No language restrictions were applied. In addition, the keywords that were combined and used in the search were: inflammation, thiophene, pyrexia and in silico studies.

## 2. The Role of Thiophene Derivatives in Inflammation

### 2.1. Thiophene-Based Compounds Inhibitors of COX and/or LOX Enzymes

Cyclooxygenase enzymes (COX) can be found in three isoforms. COX-1, also known as constitutive, is present in normal tissues and produces prostaglandins from arachidonic acid, regulating functions such as gastric mucosa production and platelet adhesiveness [32,33]. COX-2 is present in certain tissues like the uterus, kidneys and prostate. It is an inducible enzyme, and its levels increase in case of tissue damage, such as inflammation [34]. COX-3 was discovered in 2002 and is found in the central nervous system. It may be linked to the antipyretic effect of paracetamol, but its function is still not completely understood [35,36,37].

NSAIDs (non-steroidal anti-inflammatory drugs) were developed with the aim of inhibiting COX activity, but the degree of inhibition of each isoform, i.e., COX-1 or COX-2, can vary and determines side effect profiles [37,38]. Despite the development of selective NSAIDs for COX-2, many side effects have been observed, especially in situations involving chronic use. Furthermore, there are still controversies about the physiological role of this enzyme [38].

**Figure 2:**
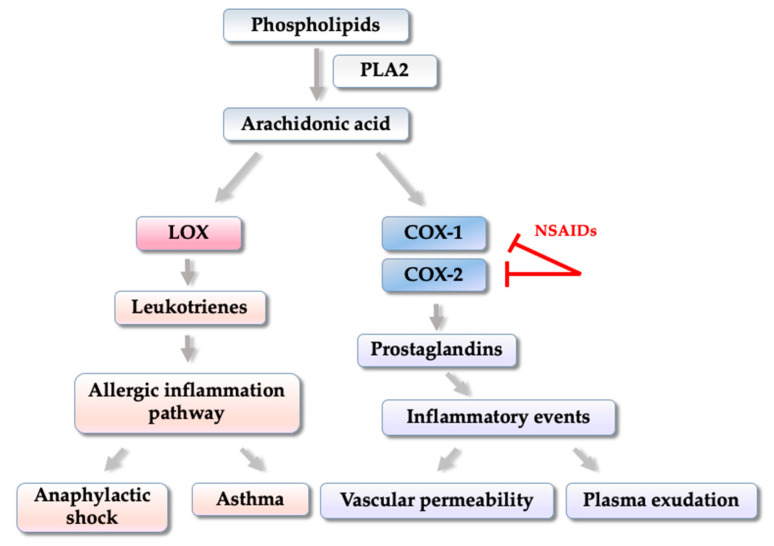
Simplified diagram of COX-1, COX-2 and LOX enzymes activation and beginning of the inflammatory process.

In inflammatory processes, activation of COX-2, and the production of prostaglandins is directly involved in inflammatory events such as increased local blood flow, increased vascular permeability, and plasma exudation (Figure 2) [39,40].

Other enzymes are involved in inflammatory processes such as lipoxygenases (LOX), which are found in several isoforms such as 5, 12, and 15, and can generate mediators called leukotrienes that promote the allergic inflammation pathway (Figure 2). These enzymes have been widely explored for being involved in pathologies such as asthma, anaphylactic shock and cardiovascular diseases. Among them, 5-LOX is the most studied because it is present in inflammatory cells (leukocytes, polymorphonuclear cells, basophils, mast cells, eosinophils, and macrophages) [41,42].

**Figure 3:**
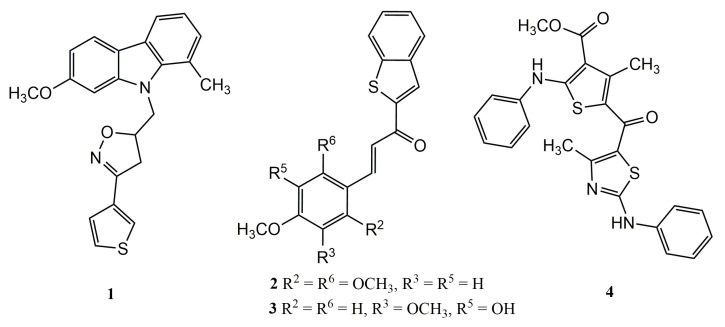
Chemical structures of thiophene-based compounds active against COX and LOX enzymes.

Many thiophenic derivatives have already been described as potential inhibitors of these important enzymes. Filali et al. [43] identified, in an in vitro study against MCF7 and HCT116 cells, that compound **1** (Figure 3) presents inhibitory activity with an IC_50_ of 29.2 µM for the 5-LOX enzyme (5-lipoxygenase). The authors stated that this potent activity was associated with the presence of methyl and methoxy radicals in its structure.

Years later, Chiasson et al. [44], searching for new molecules that inhibit the 5-LOX enzyme, evaluated a series of hybrid compounds containing benzothiophene moieties and a phenolic acid fraction, which were synthesized and tested in vitro against HEK293 cells and polymorphonuclear leukocytes. The compounds with the best activity were compounds **2** and **3** (Figure 3), which respectively presented IC_50_ values of 6.0 µM and 6.6 µM. This activity was associated with the presence of hydroxyl and methoxy groups, which were considered essential and corroborated previous results.

In an in vivo study using a guinea pig asthma model, compound **4** (Figure 3) was able to reduce inflammation when administered at 20 mg/kg (v.o.). One of the mechanisms for the action of this compound was blocking mast cell degranulation (>63%). In in vitro assays, when using compound **4** at a concentration of 100 µg/mL, inhibition of the enzyme 5-LOX was observed at approximately 57%. As with other thiophene derivatives with anti-inflammatory activity, compound **4** presents methyl, ester, and amine groups in its structure [45].

Table 1 presents the summary of the results of the thiophene-based compounds presented in this review with mechanism of action based on inhibition of COX and/or LOX enzymes.

### 2.2. Thiophene Derivatives That Modulate Gene Expression and/or Inflammatory Cytokines

Cytokines are protein molecules produced by many cell types upon antigenic stimulus that carry stimulatory, modulatory or even inhibitory signals to different immune system cells. They can act either in the cell that produced them (autocrine), in nearby cells (paracrine), or in distant cells with the aid of the bloodstream (endocrine) [46].

Cytokines are important for generating an inflammatory response at infected and/or injured sites. They can regulate gene transcription of other cytokines and stimulate increased production through signaling cascades by second messengers. The result of this stimulus may lead to formation of cytokines that increase (pro-inflammatory) or attenuate (anti-inflammatory) inflammatory processes. We have noted both pro-inflammatory: interleukins (IL) 1, 2, 6, 7, and TNF (tumor necrosis factor), and anti-inflammatory IL-4, IL-10, IL-13 and TGFβ (transforming growth factor β) cytokines [47,48].

**Figure 4:**
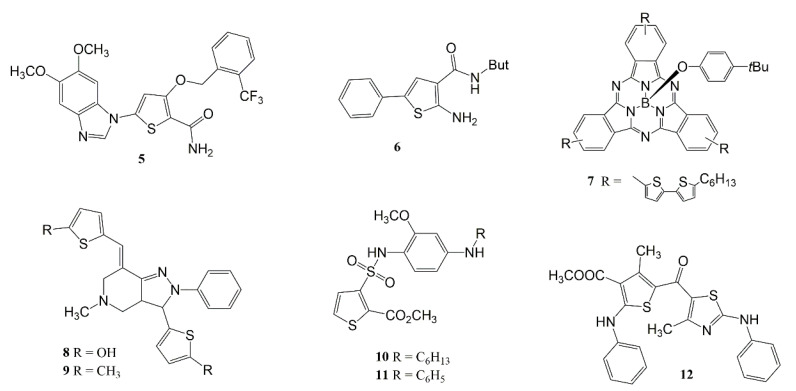
Chemical structures of thiophene-based compounds, which modulate gene expression and/or inflammatory cytokines.

For years, researchers have been studying thiophene derivatives with anti-inflammatory activity and their mechanisms of action. Hu et al. [49] evaluated methoxy-substituted thiophene derivatives (compound **5**) (Figure 4). After LPS-induced inflammation tests with THP-1 monocytes, it was observed that compound **5** was able to negatively regulate the expression of TNF-α and IL-8, and also inhibit activation of ERK, p38, and NF-ĸB (at 10 µM) [49].

In the study conducted by Ma et al. [50] the association of 2-aminothiophene (compound **6**) with platelet-derived extracellular vesicles (PEVs), was able to reduce pneumonia in a mouse model with acute lung injury (ALI), suggesting both a new technique for targeting and treating inflammation and a new scaffold for treatments against COVID-19 [50].

Eleftheriadis et al. [51] performed analyses of various thiophene derivatives based on Substitution Oriented Screening (SOS), focused on generating a compound with anti-inflammatory potential. Compound **7** (Figure 4) (based on its chemical structure) was the product chosen. According to the authors, the substitution is position 5 of the thiophene ring, as well as the presence of a 2-amino radical was important for its pharmacological activity. In computational studies of ligand efficiency, it was observed that compound **7** was able to inhibit LOX isoform (15-LOX-1) with a better efficiency than an already known inhibitor (PD-146176). Furthermore, after performing ex vivo tests on lung slices, it was demonstrated that compound **7** (at 50 mM) was able to reduce pro-inflammatory gene expression for IL-1β, IL-6, IL-8, IL-12, TNF-α, and iNOS.

Compound **8** (Figure 4) is a thiophene derivative which in in vitro anti-inflammatory activity assays on light-induced macrophages presented a better response than salicylic acid. Its effect was dose dependent (at 50 and 100 µg/mL). A possible mechanism of action was proposed involving the presence of various aromatic rings in its chemical structure, and reduction of pro-inflammatory gene expression such as TNF-α and IL-6 [52].

In in vitro assays on human red blood cells, thiophene derivatives 9 and 10 (Figure 4), at a concentration of 2 nM, were able to inhibit pro-inflammatory cytokines TNF-α, IL-1β, and IL-6, while increasing the anti-inflammatory cytokine IL-10, presenting better activity than the standard drug indomethacin [53].

Naruhn et al. [54] synthesized the thiophene derivative compound **11** (Figure 4) using compound **12**, a PPAR inhibitor (Peroxisome proliferator-activated receptor) as a prototype (Figure 4). PPAR is a receptor involved in activation of pro-inflammatory genes. Compound **11**, in concentrations equal to compound **12**, was able to reduce the transcriptional activity induced by this receptor, and negatively regulate expression for its synthesis. Upon determining the IC_50_, it was observed that compound **11** was about three times more potent than compound **12**, the respective values being 93 nM and 310 nM. The only structural difference between the two compounds is the substitution of the phenyl radical (present in 12), with a hexyl radical (in 11). This suggests that the greater flexibility or length of the hexyl chain is an essential factor towards increasing the activity of this derivative.

Patil et al. [45] evaluated new thiazolo-thiophene derivatives in an asthma model, and found that compound **4** (Figure 2) was able to significantly reduce the levels of pro-inflammatory cytokines (TNF-α, IL-1β, and IL-6) observed in bronchoalveolar lavage.

The summary of the results of the thiophene-based compounds with mechanism of action associated with gene expression and/or inflammatory cytokine modulations are present in Table 2.

### 2.3. Thiophene Derivatives with In Vivo Anti-Inflammatory Activity in Classic Models of Inflammation

Carrageenan-induced paw edema is a classic model of inflammation. The model allows observing inflammatory parameters related to neutrophil activation, pro-inflammatory mediators release (such as IL-6, IL-1β, and TNF-α), and enzymes linked to inflammation such as COX-2 [55]. Carrageenan-induced paw edema involves three stages. The first can be seen at the beginning of the inflammatory process in the first 90 min, where there is the release of histamine and serotonin, responsible for vasodilation and increased vascular permeability. In the second stage, which occurs between 90 and 150 minutes, there is biosynthesis of prostacyclins and other inflammatory process mediators. In the third stage, which occurs after 150 min, prostaglandins are synthesized in the inflamed tissue and leukocyte infiltration commences [56].

Another widely used model is ovalbumin induced asthma, this consists of triggering a classic cellular response via T helper 2 (Th2) cells and the release of inflammatory interleukins (IL) along with macrophages, eosinophils, and mast cells [57,58].

**Figure 5:**
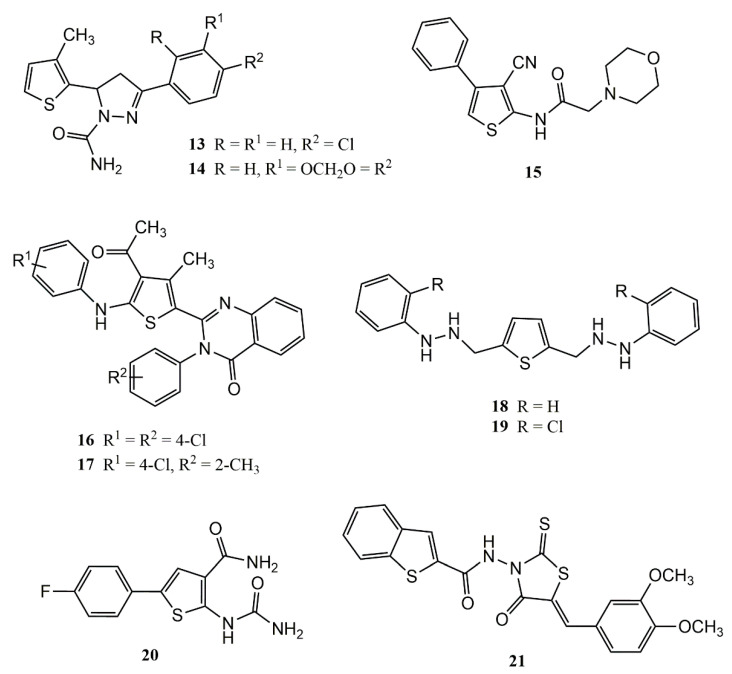
Chemical structures of thiophene-based compounds with anti-inflammatory properties in classic models of inflammation.

Some years later, Kumar et al. [59] performed in vitro tests with a series of thiophene derivatives and found that the presence of chlorine and methyl groups in the chemical structures, as observed with compounds **13** and **14** (Figure 5) is fundamental for significant PLA2 inhibition.

In a carrageenan-induced paw edema model, the study authors observed that compound **15** (Figure 5), at a dose of 50 mg/kg, presented anti-inflammatory-inhibition activity (58.46%) superior to indomethacin (47.73%). The authors associated this activity with the morphine ring coupled at the 2-amino position of the thiophene ring [60].

Compounds **16** and **17**, thiophenic derivatives both presenting methyl and chlorine substituents displayed anti-inflammatory activity comparable to sodium diclofenac in a model of paw edema induced by carrageenan (Figure 5). These derivatives were able to reduce the inflammatory process by 48.94% and 47%, respectively [61].

Gaines et al. [62] performed in vivo tests (carrageenan-induced paw edema model) with symmetric thiophene and furan derivatives (bioisosteres) as CXCR4 receptor antagonists (inflammation in Irritable Bowel Disease). The authors found that compounds **18** and **19** (Figure 5) substituted in positions 2 and 5 were the most active, with respective inhibition values of 30% and 5%.

Ligacheva et al. [63] seeking to identify ĸβ-kinase inhibitor capable of reducing Th1 (paw edema model) and Th2 (ovalbumin-induced immediate local hypersensitivity) responses in mice, noted that 2-amino-thiophenic derivatives rich in electron donor clusters, such as compound **20** (Figure 5), contain fluorophenyl, amide and carbamate groups. The evaluated compound reduced edema in a similar way to (diclofenac), and was able to reduce the edema by 1.3 times as compared to 1.6 times for diclofenac.

In the study reported by El-Miligy and coworkers [64], the authors planned and performed synthesis of hybrid compounds containing a benzothiophene (with rhodamine) as potential dual COX-2/5-LOX inhibitors. In vivo studies with COX/LOX enzymes revealed that compound **21** (Figure 5) exhibited higher COX-2 inhibition than celecoxib (IC_50_ values of 0.67 and 1.14 µM), and higher LOX inhibition than sodium meclofenamate (IC_50_ values of 2.33 and 5.64 µM). In vivo anti-inflammatory screening revealed that compound **21** was more effective in the formalin-induced paw edema assay, and safer (gastric ulcerogenic activity) than celecoxib the reference drug.

The summary of the results of active thiophene-based compounds in classic models of inflammation presented in this review are shown in Table 3.

### 2.4. In Silico Studies Involving Thiophene-Based Compounds with Anti-Inflammatory Properties

The search for new bioactive compounds is a long process, which takes several years, and which gradually becomes expensive, costing tens of millions of dollars before reaching the goal of having a new chemical entity capable to be used in therapy. Aiming to reduce these costs, reducing the number of molecules that need to be synthesized, reducing the number of compounds that effectively need to be tested in the various in vitro, ex vivo, and in vivo assays, several computational methods, also known as in silico methods, or CADD (Computer-Aided Drug Design) studies were developed and have been constantly improved in order to reduce cost and times of the drug design and discovery process, and increase the chances of success. In silico methods are increasingly being used in both industry and in universities. They involve understanding molecular interactions from both a qualitative and quantitative point of view-based in mathematical tools. These methods generate and manipulate three-dimensional (3D) molecular structures, calculate descriptors and dependent molecular properties (pharmacokinetic (ADME) and toxicity properties, among others), model constructions, and employ other computational drug research tools. Analysis of the molecular structure of a given system allows relevant information to be extracted, as well as predicting the potential of the bioactive compound [65,66,67].

In silico methods are subdivided into two major general types of CADD approaches: Structure-Based Drug Design (SBDD) and Ligand-Based Drug Design (LBDD). SBDD methods are used to help investigators to predict, with precision and efficiency, in three dimensions, at a molecular level, the position (affinity) of small molecules (drug candidates or ligand) to molecular targets, usually proteins (enzymes) and nucleic acids (DNA and RNA). Where molecular docking is one of the most widespread and most used tool. In addition, LBDD approaches have been used when only the structure of the ligands are known, or when the data on the biological activities of those ligands were known. LBDD allows determinate the correlations between chemicals structures and the physicochemical properties of ligands and their biological activities. The three main categories of LBDD are: (a) pharmacophore models, which make it possible to identify the essential characteristics of the ligands for maintenance of biological activity; (b) Quantitative Structure–Activity Relationships (QSAR), which results in quantitative activity data based on the physicochemical properties of the ligands; and (c) similarity searching, which helps to predict the biological activity and the physicochemical properties of ligands based on existing data from other ligands with similar chemical structures [65,66,67].

**Figure 6:**
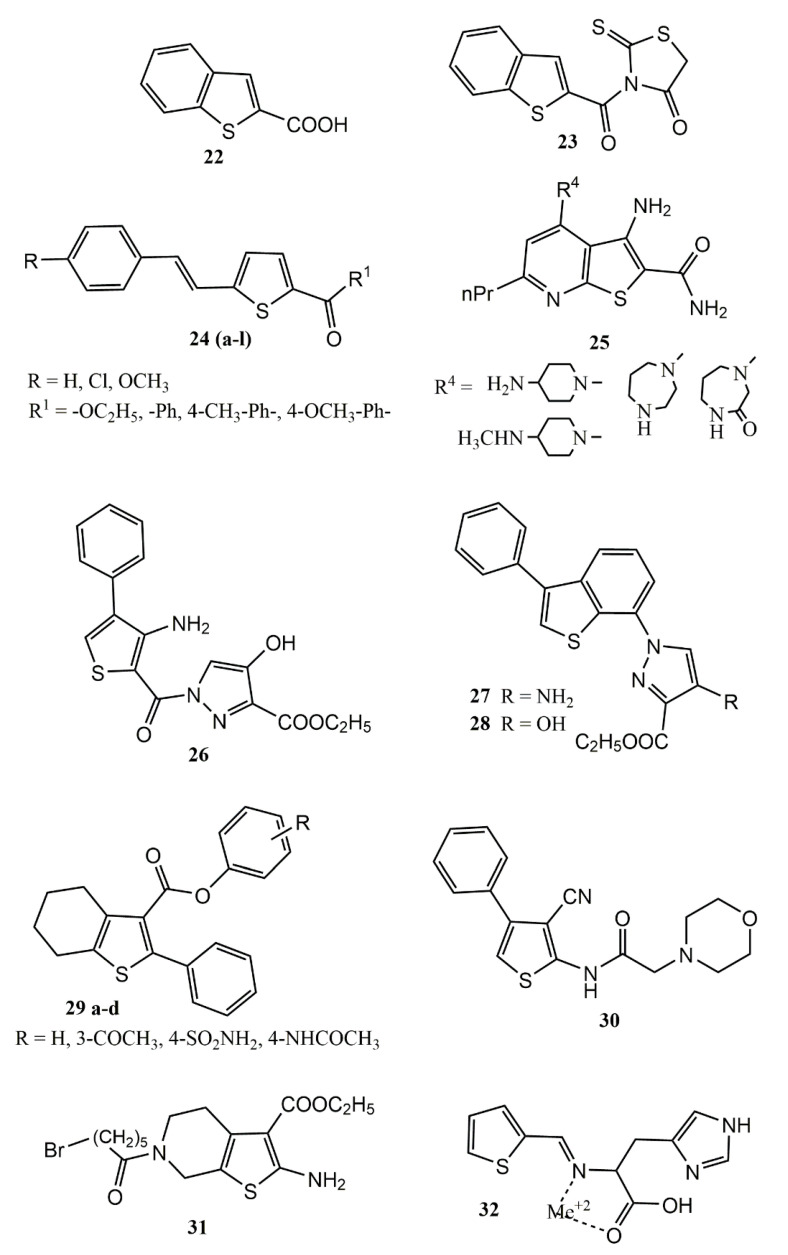
Chemical structures of thiophene-based compounds with anti-inflammatory properties in in silico studies.

In this context, Sagaama and Issaoui [68] performed a theoretical study involving molecular geometry, vibrational, pharmaceutical (^1^H and ^13^C Nuclear Magnetic Resonance (NMR) and UV-vis spectrum), and electronic properties (TD-DFT (time-dependent density-functional theory), HOMO-LUMO transitions (highest occupied molecular orbital and lowest unoccupied molecular orbital)), Hirshfeld surfaces, and molecular docking, using as a prototype 1-benzothiophene-2-carboxylic acid (2BT) (22) (Figure 6). Molecular docking was carried out using the iGEMDOCK program and Discovery studio software, against several targets, including: Human Immunodeficiency Virus type 1 (HIV) (PDB id: 1DLO), Bat SARS-like coronavirus (6LU7) (PDB id: 6LU7), and the inflammatory targets COX-2 (PDB id: 3LN1) and 5-LOX (PDB id: 3V92). The binding energy for COX-2 and 5-LOX, were respectively −81.44 and −72.48 kcal/mol.

Molecular docking with a hybrid compound (**23**) containing 2BT and rhodamine (Figure 6) was performed against the COX-2 and 5-LOX enzymes, and it was revealed that the hybrid presents increased interaction energy against these inflammatory targets with respective binding energies of −98.37 and −91.07 kcal/mol. The studies suggest that (2BT) (22), and especially the hybrid 2BT+rhodamine (23), are potential competitive dual inhibitors COX-2/5-LOX, and can be used as prototypes for development of new anti-inflammatory drugs.

Karthick, Balachandran and Perumal [69] also performed spectroscopic investigations with compound **22** (Uv-vis spectra and FT-IR (Fourier-transform infrared)), intra and intermolecular interactions, and molecular docking (Figure 6). To evaluate anti-inflammatory potential, molecular docking studies were carried out using SWISSDOCK webserve, against COX-2 (PDB id: 1CX2) and Prostaglandin H2 synthase (PDB id: 1PTH). Although the binding affinity value were low, the authors confirmed the potential anti-inflammatory activity of compound **22**, which presented binding affinity values of −6.13 kcal/mol (for COX-2), and −6.28 kcal/mol (for Prostaglandin H2 synthase).

Molecular docking was performed on COX-2 (PDB id: 3LN1) and 5-LOX (PDB id: 3V99) enzymes using the software Molecular Operating Environment, and it was observed that compound **22** (Figure 6) displayed significant dual COX-2/5-LOX inhibitory activities with respective binding affinities of −12.47 and −11.79 kcal/mol. In silico physicochemical and pharmacokinetics properties were also predicted using the Molinspiration online property calculation toolkit, MolSoft software, the PreADMET calculator, and the Osiris property explorer. Compound **22** passed well in all filters (ADMET, physicochemical, and drug-like properties) demonstrating lead compound potential for dual COX-2/5-LOX inhibitor development.

Singh et al. [70] synthesized a series of novel thiophene derivatives, and performed molecular docking studies with COX-2 (PDB id: 1CX2) using QUANTUM 3.3.0 software. Compound **24** (a-l) (Figure 6) presented binding energy values comparable to conventional NSAIDs, with values ranging between −6.23 and −6.32 kcal/mol.

Ul-Haq et al. [71] analyzed a group of 46 thienopyridine derivatives (25) (Figure 6) to identify the structure required to inhibit kappa B kinase subunit β (IKKβ). IKKβ is a target for treatment in cancer and inflammatory diseases. Molecular docking studies on IKKβ (PDB id: 4KIK), using DFT, 3D-Quantitative Structure Activity Relationship (3D-QSAR), Comparative Molecular Field Analysis (CoMFA) and Comparative Molecular Similarity Indices Analysis (CoMSIA) were performed, and the authors identified that inhibitory potential against IKKβ is increased with radicals in position 4 presenting electron withdrawing hydrophobic groups. These findings will make it possible to design new IKKβ inhibitors.

El-Shoukrofy and coworkers [72] designed and synthesized a series of thiophene pyrazole hybrids to obtain new COX-2 inhibitors. In vitro COX-1 and COX-2 enzymatic inhibition assays were performed, with in vivo evaluation of anti-inflammatory activity through formalin-induced paw edema bioassays in models of acute and sub-acute inflammation, with evaluation of ulcerogenic activity as well. In parallel, chemoinformatics studies were carried out to evaluate ADME properties and pharmacokinetic profiles using the Molinspiration tool and the PreADMET calculator. Drug likeness was predicted using Molsoft software, and molecular docking in COX-2 (PDB id: 3LN1) was performed using Molecular Operating Environment (MOE) version 2014.09. In the in vitro studies, all of the compounds presented higher selectivity for COX-2 than for COX-1. In the in vivo studies, some of the compounds studied presented greater anti-inflammatory activity than celecoxib, and with fewer gastrointestinal effects. In the in silico studies, some hybrids (26–28) (Figure 6) presented better drug-likeness and ADMET profiles. Docking studies demonstrated that half of the compounds (including 26–28) presented activity equal to or greater than the co-crystallized ligand (celecoxib). Taken together, these results indicate that thiophene pyrazole hybrids are promising anti-inflammatory drug candidates with moderate and selective COX-2 inhibition.

Khatri and colleagues [73] conducted a similar study, using in vitro, in vivo, and in silico studies with 2-phenyl-4,5,6,7-tetrahydro[*b*]benzothiophene derivatives as selective COX-2 inhibitors. Compounds (**29a–d**) (Figure 6) were revealed as selective COX-2 inhibitors, presenting IC_50_ values in the 0.31–1.40 µM range. In the carrageenan-induced paw edema assay, the compounds **29a–d** displayed potent anti-inflammatory activity superior to celecoxib. To confirm the selectivity of the compounds as potential COX-2 inhibitors, molecular docking was performed using Schrödinger’s GLIDE program against COX-1 (PDB ID: 1N8Z) and COX-2 (PDB ID: 3LN1). The molecular docking studies corroborated the in vitro results. Binding of the compounds was not observed at the COX-1 active site, due to the constrained binding pocket. However, they docked very well at the COX-2 active site, with similar orientations, and interaction energy values that were equal to Celecoxib, thus demonstrating the observed selectivity.

Helal et al. [60] performed synthesis, quantum-chemical modeling, and evaluation of a series of 2-amino-thiophene derivatives for anti-inflammatory activity. Molecular modeling studies using the semi-empirical Method PM3 were performed, and Method PM3 was also used to calculate molecular and electronic parameters such as electronegativity, bond lengths and angles, chemical potential, global hardness and softness, dipole moment, total energy, electronic energy, binding energy, and HOMO and LUMO energies. The anti-inflammatory activity was evaluated using the carrageenan-induced paw edema assay, and most of the evaluated derivatives presented moderate activity comparable to indomethacin, the reference drug. Compound **30** (Figure 6) (with a morpholine ring) was the most active of the series, presenting anti-inflammatory activity superior to that of indomethacin, it is now considered a promising hit compound (for further modification) to obtain new and powerful NSAIDs.

In a recent study by our group [26], we synthesized the thiophene derivative **31** (Figure 6), an analog of the NSAID tinoridine. Antinociceptive and antipyretic activities were evaluated in vivo at doses of 50 and 100 mg/kg, and compound **31** was able to significantly reduce the number of abdominal contortions in the acetic acid induced abdominal contortions test, as well as the licking time in the first and second phases of the formalin-induced nociception test. Compound **32** also significantly increased the latency time in the hot plate test, and reduced pyrexia at 30, 60, and 120 min. Molecular docking was carried out using Molegro Virtual Docker program with COX-1 (PDB ID: 4O1Z) and COX-2 (PDB ID: 4M11) as targets. The docking revealed MolDock Score values of −106.93 kcal/mol (for COX-1), and −110.84 kcal/mol (for COX-2), indicating that compound **32** is a potential anti-inflammatory agent, though likely as a non-selective COX inhibitor.

Jonh and coworkers [74] synthesized thiophene-2-carboxaldehyde-*L*-histidine (thio-*L*-his) and its metal complexes (Co (II), Ni (II), Cu (II) and Zn (II)) 32 (Figure 6), and evaluated their pharmacological properties (including anti-inflammatory activity) while performing molecular docking and DFT studies. The anti-inflammatory activity was evaluated in vitro in an albumin denaturation model, and it was observed that thio-*L*-his and its complexes exhibit inhibitory effects in a dose-dependent manner on heat-induced albumin denaturation. The complex Cu (II)-(thio-*L*-hi) was the most effective, causing inhibition of up to 80.11%, superior to the activity of aspirin (76.89%). Molecular docking was performed using AutoDock 4.2 software, and the crystal structure of the COX-2 enzyme (PDB id: 4C0X), (related to inflammation). The Cu (II) and Zn (II) complexes presented the highest binding energy values, respectively −7.42 and −7.22 kcal/mol, being the best potential inhibitors of this important enzyme.

A summary of the results of the in silico studies of thiophene-based compounds with potential anti-inflammatory activity is shown in Table 4.

## 3. Conclusions

**Figure 7:**
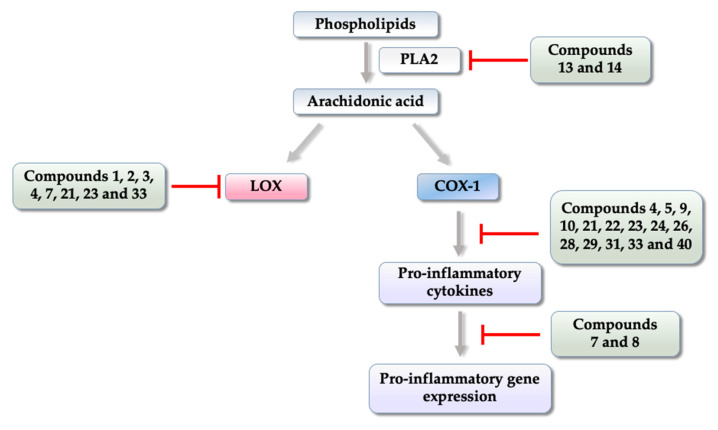
Representative figure of the general mechanisms of action of the thiophene-based compounds presented in this review.

This review demonstrates the importance of thiophene-based compounds as privileged structures in drug design and in discovery of novel anti-inflammatory agents. The vast majority of their planned and synthesized derivatives present anti-inflammatory activity superior to the reference NSAIDs as was shown in in vitro, and in silico, and in vivo assays. In Figure 7, we schematically represent the summary of the main mechanisms of action of thiophenic derivatives presented in this work, demonstrating where each one interferes within the inflammatory cascade, thus enabling its anti-inflammatory effects.

Many studies reveal structural characteristics important for the anti-inflammatory activity of thiophene derivatives. Although it is difficult to generalize due to the great diversity of the structures, certain substitution patterns are frequently described, such as the presence of free or substituted amines, amides at C-2, the presence of carboxylic acid derived groups (acids, esters, and nitrile) at C-3, and the presence of at least one phenyl ring substituted with radicals, methyl, methoxy, or chlorine at C-4 and C-5. The importance of these groups/radicals for anti-inflammatory activity and for biological target recognition, especially for COX and LOX inhibition, is highlighted.

It was found that the vast majority of in silico studies use molecular docking to validate the results of anti-inflammatory activities discovered during in vitro and/or in vivo assays, or to perform virtual screening of potential ligands for selected targets. This includes evaluating the selectivity of the derivatives herein presented against different COX isoforms. Of the most extensively evaluated targets we included the two main isoforms of cyclooxygenase (COX-1 and COX-2), and the 5-LOX enzyme.

## Figures and Tables

**Table 1:** Summary of the thiophene-based compounds acting as COX and/or LOX inhibitors.

Model	Mechanisms	Reference
In vitro assay with soybean lipoxygenase	5-LOX inhibitory activity	[43]
In vitro assay with modified HEK293 cells	5-LOX inhibitory activity	[44]
Ovalbumin-induced airway inflammation in Guinea pig	Blocking mast cell degranulation	[45]
In vitro assay with lipoxygenase enzyme	5-LOX inhibitory activity	[45]

**Table 2:** Summary of the thiophene-based compounds that modulate gene expression and/or inflammatory cytokines.

Model	Mechanisms	Reference
In vitro essay by qRT-PCR and ELISA	Inhibition of pro-inflammatory cytokines expression (TNF-α, IL-8, ERK, p38, and NF-ĸB)	[49]
Mouse with acute lung injury	Association with platelet-derived extracellular vesicles	[50]
In silico essay with Substitution Oriented Screening model and in vitro essay with lipoxygenase enzyme	LOX inhibition	[51]
Ex vivo essay in precision-cut lung slices	Inhibition of pro-inflammatory gene expressions (IL-1β, IL-6, IL-8, IL-12, TNF-α, and iNOS)	[51]
In vitro essay by ELISA and qRT-PCR with light-induced macrophages	Reduction of TNF-α and IL-6 expressions	[52]
In vitro essay by ELISA	Inhibition of TNF-α, IL-1β and IL-6 activities and activation of IL-10	[53]
In vitro essay by TR-FRET	Reduction of PPAR transcriptional activity	[54]
Ovalbumin-induced airway inflammation in Guinea pig in association with ELISA method	Reduction of TNF-α, IL-1β and IL-6 cytokines	[45]

**Table 3:** Summary of the active thiophene-based compounds in classic models of inflammation.

Model	Mechanisms	Reference
In vitro essay with Phospholipase A_2_ from *Vipera russelli*	Inhibition of PLA2	[59]
Carrageenan-induced paw edema in Albino rats	Decreasing the paw volume after carrageenan administration	[60]
Carrageenan-induced paw edema in rats	Decreasing the paw volume after carrageenan administration	[61]
Carrageenan-induced paw edema in mouse	CXCR4 receptor antagonists	[62]
Paw edema and ovalbumin-induced immediate local hypersensitivity	IKK-2 inhibition to reduction of Th1 response	[63]
In vitro essay on COX/LOX enzymes	COX-2 and LOX inhibition	[64]
Formalin-induced paw edema and Gastric ulcerogenic activity	COX-2 and LOX inhibition	[64]

**Table 4:** Summary of the predictions from in silico studies performed with thiophene-based compounds with potential anti-inflammatory activity.

Model	Mechanisms	Reference
Theoretical and DFT studies, and Molecular docking	Dual inhibition of COX-2/LOX-5	[68]
Molecular docking	Dual inhibition of COX-2/LOX-5 and Prostaglandin H2 synthase inhibition	[69]
Molecular docking	COX-2 inhibition	[70]
DFT, QSAR, CoMFA, CoMSIA and Molecular docking	IKKβ inhibition	[71]
ADMET predictions and Molecular docking	COX-2 inhibition	[72]
Molecular docking	COX-2 inhibition	[73]
Molecular parameters by PM3 method	Decreasing the paw volume after carrageenan administration	[60]
Molecular docking	Non-selective COX-1 and COX-2 inhibition	[26]
DFT and Molecular docking	COX-2 inhibition	[74]

## Data Availability

Data sharing not applicable.
